# Dealing with a new disease

**DOI:** 10.1007/s12471-022-01727-2

**Published:** 2022-10-24

**Authors:** Y. M. Pinto

**Affiliations:** Department of Experimental Cardiology, Amsterdam University Medical Centres, location Academic Medical Centre, University of Amsterdam, Amsterdam, The Netherlands

In this issue of the *Netherlands Heart Journal*, a number of papers describe the cardiovascular impact of the coronavirus disease 2019 (COVID-19) pandemic [1, 2]. These studies highlight how this novel disease triggers an impressive worldwide response by both healthcare workers and scientists. If anything, such papers highlight the importance of learning from looking back, while appreciating how it shows the sheer impossibility to look forward.

When COVID-19 initially emerged, a severe infection with severe acute respiratory syndrome coronavirus 2 could surprise everyone concerned with the patient’s team with some severe, yet unexpected clinical complications the disease presented. For instance, already in early April 2020, Faggiano et al. described cases of acute pulmonary embolism in COVID-19 [3]. In the first weeks of the pandemic, many patients succumbed to pulmonary embolisms that were either unrecognised or recognised too late. Now, the procoagulant properties of this infection are well-known, and prevention of thrombo-embolism is part of the routine approach. This illustrates that—as with all medical progress—increasing knowledge rapidly translates into better and often even more efficient care.

The pandemic had a profound effect on gaining and spreading knowledge. The rapid ways to publish accepted manuscripts online and to place even not yet accepted manuscripts online, for example on medRxiv, have certainly been pivotal to disseminate new ideas, findings and insights at an unprecedented pace. It may indeed be the first pandemic where the spread of the virus itself was rivalled by the rapid and often similarly overwhelming spread of scientific discoveries. It has even been suggested that the pandemic has changed the very nature of scientific publishing [4], with a rush towards preprints and unprecedented rapid refereeing of submitted papers to share new insights as quickly as possible.

Taken together, we now face the relative comfortable perspective of being able to look back. In this issue of the journal, papers reflect on the longer-term effects of COVID-19 and on its effects on the delivery of care. This provides a good time to take the readers of our journal back to these early days of the pandemic, when we were facing a completely novel disease, as this underscores that a global health crisis as this one was for a large part a knowledge crisis.
